# Publication trends of research on conjunctival melanoma during 1997–2022: A 25-year bibliometric study

**DOI:** 10.3389/fonc.2022.960494

**Published:** 2022-08-18

**Authors:** Wei Xu, Ludi Yang, Shengfang Ge, Shichong Jia, Fen Gu

**Affiliations:** ^1^ Department of Ophthalmology, Shanghai Key Laboratory of Orbital Diseases and Ocular Oncology, Shanghai Ninth People’s Hospital, Shanghai Jiao Tong University School of Medicine, Shanghai, China; ^2^ Tianjin Eye Hospital, Tianjin Key Lab of Ophthalmology and Visual Science, Nankai University Affiliated Eye Hospital, Tianjin Eye Institute, Tianjin Key Laboratory of Ophthalmology and Visual Science, Tianjin, China; ^3^ Department of General Surgery, Shanghai Ninth People’s Hospital, Shanghai Jiao Tong University School of Medicine, Shanghai, China

**Keywords:** conjunctival melanoma, publication trends, bibliometric analysis, targeted therapy, immunotherapy

## Abstract

**Background:**

Conjunctival melanoma (CM) is a life-threatening ocular tumor with a high rate of local recurrence and metastasis. Our objective is to analyze research trends in CM field and compare contributions from different countries, institutions and authors.

**Methods:**

We extracted all CM-related publications published from 1997 to 2022 from the Web of Science database and applied Microsoft Excel and VOSviewer to review publication data, analyze publication trends, and visualize relevant data.

**Results:**

A total of 708 publications were identified. The United States contributed the most publications (280) and citations (8,781 times) with the highest H-index value (47). The *Ophthalmic Plastic and Reconstructive Surgery*, *British Journal of Ophthalmology*, *American Journal of Ophthalmology* and *Cornea* were the most productive journal concerning CM, and Shields CL, Shields JA, Jager MJ as well as Finger PT had published the most papers in the field. Keywords were classified into three clusters: clinical research, management-related research and genetic research. The keywords “primary acquired melanosis”, “metastasis” and “*BRAF* mutations” were most frequently emerged. According to the average appearing year (AAY), targeted therapy (AAY of 2019.0) and nivolumab (AAY of 2018.7) were identified as the main focuses of the field in the near future.

**Conclusion:**

In the past 25 years, the United States, Germany, England and the Netherlands held the leading position in the CM research. A group of scholars made important contributions to CM research and will continue to guide cutting-edge research. Treatments that have been shown to be effective for advanced cutaneous melanoma, such as targeted therapy and immunotherapy, are potential focuses for future CM research.

## Introduction

Conjunctival melanoma (CM) is a potentially deadly ocular tumor which originates from melanocytes in the basal layers of the conjunctiva, accounting for 2% of all eye malignancies (1). The reported annual incidence was 0.3–0.8/million in Caucasian adults, and the incidence is still increasing (2–4). Tumors confined to the conjunctiva are usually treated by surgical excision plus cryotherapy, while advanced diseases like deeply invasive tumors may require more extensive treatment (5). Despite surgical removal of the tumor, over half of CM patients may develop local recurrence, and over one third of patients will die of the disease within 10 years (3, 6–8). Several risk factors for worse prognosis have been demonstrated, including greater tumor thickness, non-bulbar location, low pigmentation, histologic ulceration, tumors arising *de novo* and positive sentinel lymph node (6, 9–13). Researchers are also constantly updating the American Joint Committee on Cancer (AJCC) cancer staging system for better risk stratification and prognostic assessment, and its accuracy has also been well confirmed in various populations around the world (9, 14–16). Compared with uveal melanoma (UM) which affects the choroid, ciliary body or iris, CM was found to have a different etiology and genetic background (17). The classic genetic features of CM, such as the mutations of *BRAF*, *NRAS* and *TERT*, were also identified in cutaneous melanoma (5). Analysis of epidemiology and pathogenesis also reflected differences between CM and UM, as well as similarities between CM and cutaneous melanoma (18–20).

The past few decades have witnessed many encouraging achievements in CM research. Bibliometrics is an optimal measure to evaluate particular research trends concerning a certain field over time and compare the contributions across countries, institutions and journals (21). Bibliometric methods can not only quantitatively and qualitatively analyze publications, but they can also characterize and predict the development of CM research. Here, we conducted a comprehensive study of the current state of global CM research based on Web of Science (WOS) data and explored public trends and potential focuses within this field *via* bibliometrics.

## Methods

### Search strategies and data collection

**Figure 1:**
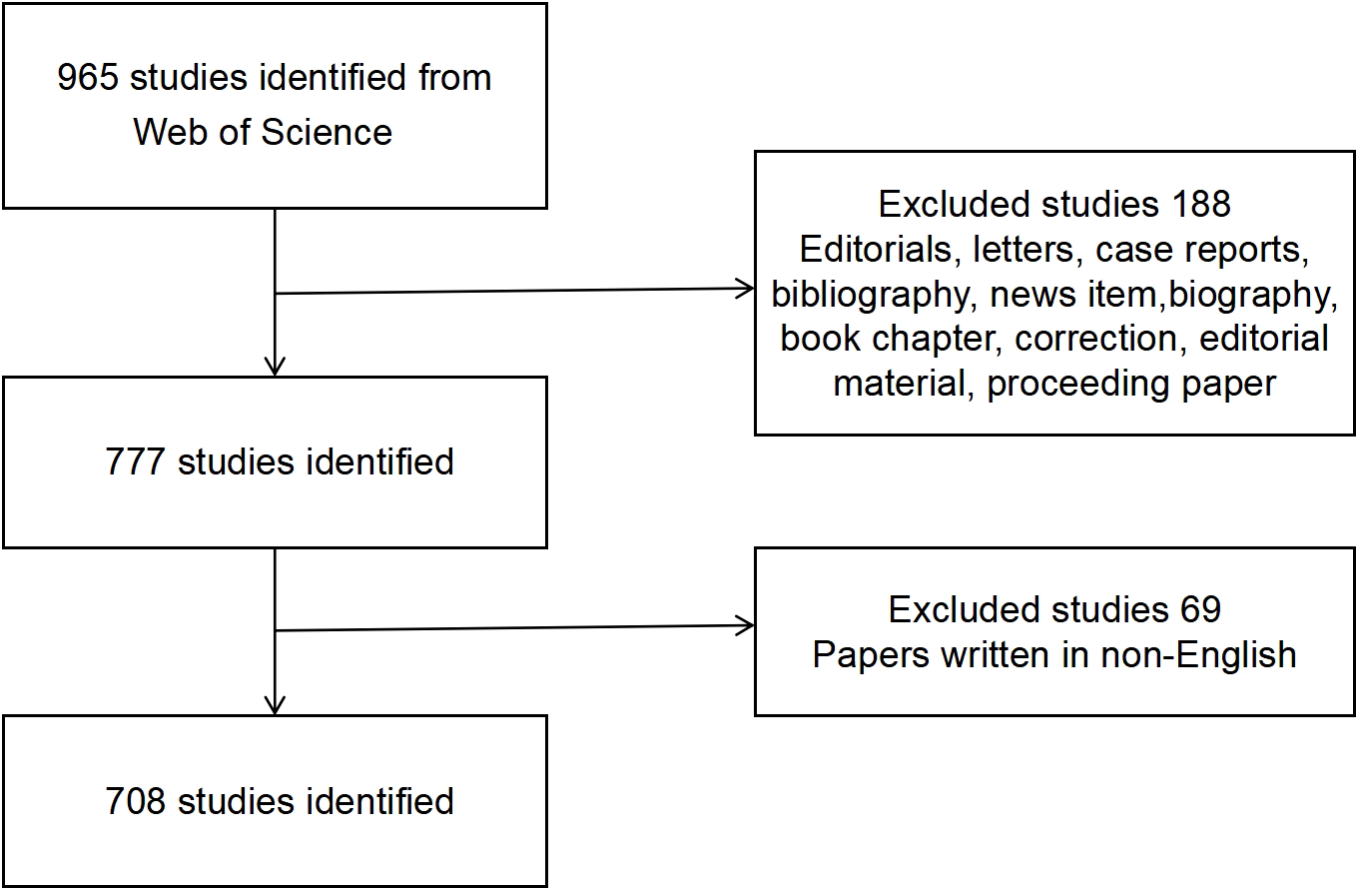
Flow diagram of CM researches inclusion process.

WOS Core Collection (WOSCC) was widely recognized as the most suitable database for bibliometric analysis. In February 2022, we conducted a literature search for the recent 25 years (1997-2022) in the WOSCC to identify relevant publications. All searches were completed on February 26, 2022 to avoid biases brought by the daily renewal of the database. To be included in this review, the keyword of manuscripts should be “TS = conjunctival melanoma”, the document type was articles or reviews and the manuscripts should be written in English. **Figure 1** exhibited our inclusion and exclusion procedures. Finally, a total of 708 publications (618 original articles and 90 reviews) were included.

The reports were reviewed for data on publication numbers, countries and regions, authors, citations, and H-indexes, from WOSCC. Microsoft Excel 2010 (Redmond, Washington, USA) and VOSviewer (Leiden University, Leiden, Netherlands) were used to record and analyze the data.

### Bibliometric analysis

We conducted bibliometric analysis from the following aspects:

#### Contribution of countries to publications

We used Microsoft Excel 2010 to identify and rank the number of publications among different countries. We also calculated relative research interest (RRI), which reflected the number of publications in a specific field divided by all publications across all fields per year, to evaluate the global attention to CM.

#### Citations and H-Index

We extracted the information concerning citations from the WOS database. The H-index, defined as a scholar, a country or an author published H papers that have been cited in other publications at least H times, is applied to evaluate the scientific research influence of a scholar, a country, or an author.

#### Growth trends of publications

To analyze future publication patterns in the field, we applied Microsoft Excel 2010 to generate the prediction model f(x) = ax^3^ + bx^2^ + cx + d based on cumulative publications. In this formula, x means time (year) and f (x) represents the cumulative volume of publications in a certain year.

#### Contribution of journals, institutions, and authors

We acquired the information related to the top journals, institutions and authors and their number of publications from the WOS database and we used Microsoft Excel 2010 to assess their contributions.

#### Analysis of keywords

VOSviewer was employed to visualize and construct the network of keywords from titles and abstracts. Keywords were categorized into disparate clusters according to co-occurrence analysis and simultaneously color-coded by time course. Furthermore, the concept of average appearing year (AAY) was adopted to assess the novelty and the time trend.

## Results

### Contribution of countries to global publications

**Figure 2:**
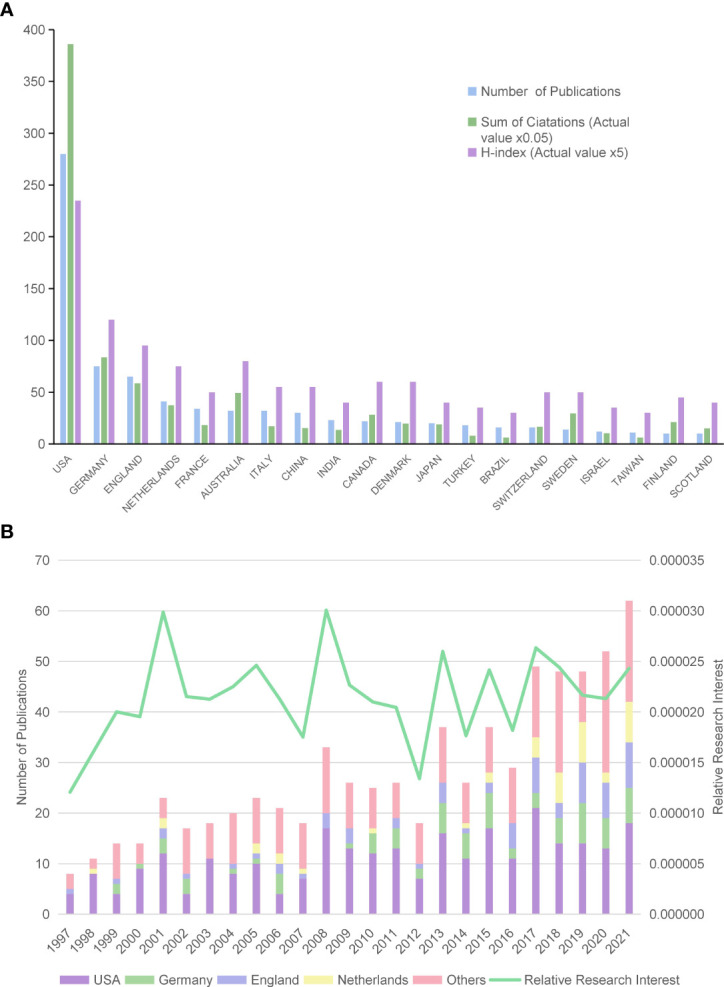
Contributions to the CM research of different countries/regions. **(A)** The number of publications, citations (×0.05), and H-index value (×5) of the top 20 countries or regions; **(B)** histogram shows the amount of publications worldwide and the top four countries. Line chart shows the time course of RRI.

Over the course of the past 25 years, as shown in **Figure 2A**, USA had the largest number of publications (n=280, 39.5%), followed by Germany (n=75, 10.6%), England (n=65, 9.2%) and the Netherlands (n=41, 5.8%). Furthermore, as for the number of publications per year, the year with the most publications was 2021 (n=62, 8.8%). In the past 25 years, the United States presented with the largest number of publications each year. Then we drew all-field publications into consideration to assess the global attention: according to RRI values, global attention in this area was below 0.0020% until 2000, fluctuated around 0.0025% over the last 20 years, and peaked at 0.0030% in 2001 and 2008 (**Figure 2B**).

### Citations and H-Index

Based on the citation report acquired from the WOS database, all publications related to CM have been cited 16,458 times since 1997 (11,629 citations without self-citations), with an average citing frequency of 23 times per paper. The most regularly cited papers were the papers from the United States, accounting for 53.4% of all citations (8,781 citations and 7,719 citations without self-citations) with an H-index of 47. Germany ranked second with a citation frequency of 1,800 (1,674 citations without self-citations) and an H-index of 24, followed by England with 1,269 citations (1,171 citations without self-citations) and an H-index of 19 (**Figure 2A**).

### Growth trends prediction

**Figure 3:**
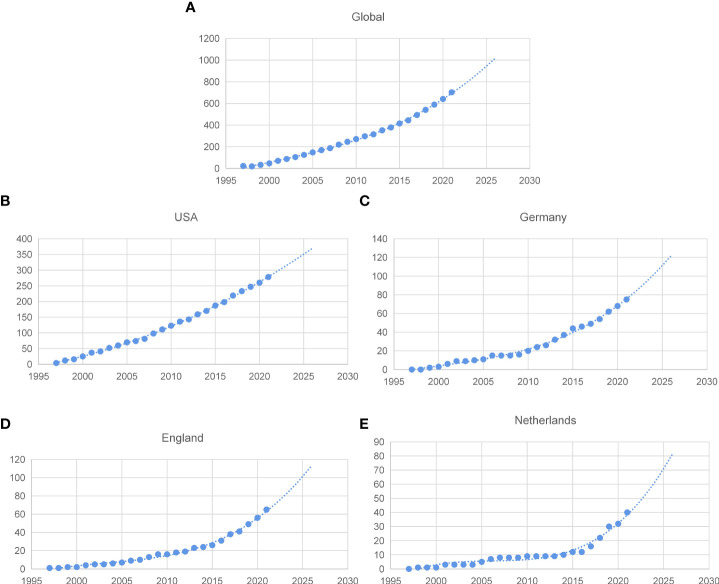
Fitting curves of publications growth trends. **(A)** Global; **(B)** the United States; **(C)** Germany; **(D)** England; **(E)** Netherlands.

Model fitting curves of the growth in CM publication demonstrated a significant correlation between time and a cumulative number of publications (**Figure 3**). Furthermore, publication trends for the following 5 years were estimated according to cumulative publication numbers over the past 25 years. The volume of global publications increased uniformly over time (**Figure 3A**), which is in accord with several major countries such as the United States and Germany (**Figures 3B, C**), while Netherlands exhibited an obviously faster growth in recent five years (**Figure 3E**).

### Journals, institutions, and authors publishing research on CM

**Figure 4:**
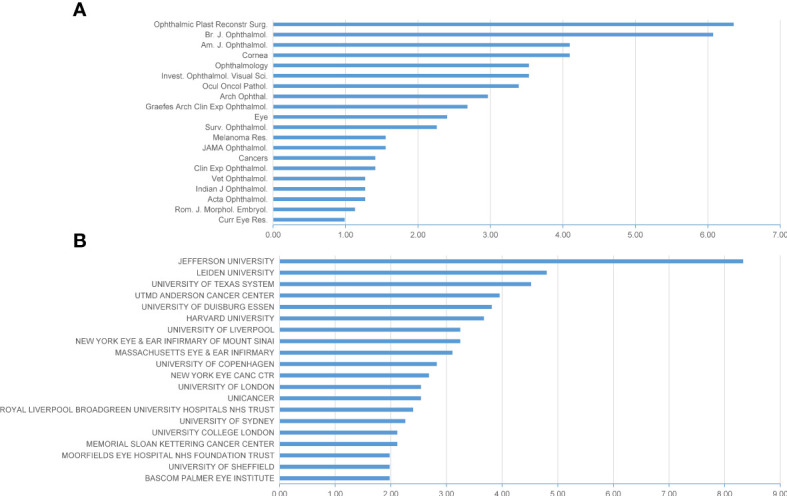
Distribution of journals and institutions focusing on CM. **(A)** Top 20 journals publishing the most papers in this field; **(B)** top 20 institutions publishing the most papers in this field.

More than half of the papers within this field were published in the 20 journals listed in **Figure 4A** (n=377, 53.3%), the *Ophthalmic Plastic and Reconstructive Surgery* published the most with 45 records (6.4%), followed by *British Journal of Ophthalmology* (43 papers, 6.1%), *American Journal of Ophthalmology* (29 papers, 4.1%) and *Cornea* (29 papers, 4.1%). Moreover, *Ophthalmology* and *Investigative Ophthalmology and Visual Science* ranked the fifth, with both 25 papers (3.5%).

When it regards to institutions, Jefferson University in the United States published the most studies (n=59, 8.3%), followed by Leiden University in the Netherlands (n=34, 4.8%), University of Texas System (n=32, 4.5%) in the United States, UTMD Anderson Cancer Center (n=28, 4.0%) the United States and University of Duisburg Essen (n=27, 3.8%) in Germany. Among the top 20 institutions identified for their publications, nine are in the United States, six are English institutions, and the other five are located in the Netherlands, Denmark, France, Germany and Australia.

The top 10 authors published a total of 147 papers, accounting for 20.1% of the total literature in this field. Shields CL of Jefferson University has published 57 papers related to CM, ranking first in the number of papers published, and her H-index is also the highest, with 24 (**Table 1**). Shields JA of Jefferson University ranked second with 51 papers. Jager MJ of Leiden University and Finger PT of the New York Eye Cancer Center each published 24 articles, tied for third place. Among the top 10 authors, six are from the United States, two from the Netherlands, and the other two from Denmark and England.

**Table 1:** Top 10 authors with the most publications in CM research.

Author	Country	Affiliation	No. of publications	No. of citations (all)
Shields CL	USA	Jefferson University	57	2229
Shields JA	USA	Jefferson University	51	2131
Jager MJ	Netherlands	Leiden University	24	271
Finger PT	USA	New York Eye Cancer Center	24	937
Esmaeli B	USA	University of Texas System	23	695
Coupland SE	England	University of Liverpool	19	571
Heegaard S	Denmark	University of Copenhagen	17	370
Eagle RC	USA	Jefferson University	16	220
Lally SE	USA	Jefferson University	16	319
Marinkovic M	Netherlands	Leiden University	16	172

### Analysis of keywords and focuses of CM research

**Figure 5:**
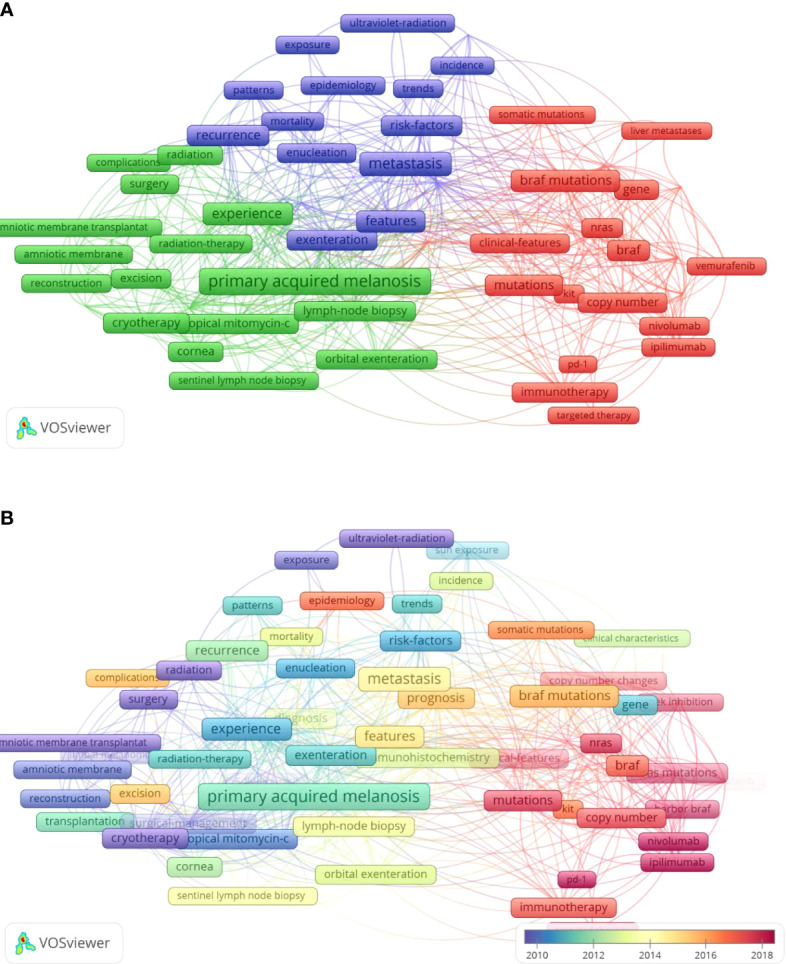
The analysis of keywords in CM researches. **(A)** Mapping of the keywords in CM researches. All keywords were divided into three clusters and given different colors: management-related research (left in green), genetic research (right in red), and clinical research (up in blue); **(B)** distribution of keywords based on the average time of appearance. Red indicates recent appearance while purple indicates early appearance.

Keywords that appeared more than five times in the titles and abstracts of 708 publications were analyzed using VOSviewer. We identified 64 keywords after merging words with the same meaning words and excluding meaningless words, and categorized them into three clusters: (1) clinical research, (2) management-related research, and (3) genetic research (**Figure 5A**). In the clinical research cluster, the most frequently mentioned keywords were metastasis (66 times), features (35 times), recurrence (28 times) and prognosis (28 times). As for the management-related research cluster, primary acquired melanosis (96 times), experience (45 times), cryotherapy (22 times) and lymph-node biopsy (20 times) were the primary keywords. As with the genetic research cluster, the common keywords were *BRAF* mutations (38 times), mutations (27 times), *NRAS* mutations (18 times) and immunotherapy (17 times).

On the other hand, we colored keywords according to the AAY by VOSviewer (**Figure 5B**). Keywords that appear relatively early are marked in blue, while those that appear more recently are marked in red. Keywords such as targeted therapy (cluster 3, AAY of 2019.0) and nivolumab (cluster 3, AAY of 2018.7) have emerged recently, while cryotherapy (cluster 2, AAY of 2009.4) and 5-fluorouracil (cluster 2, AAY of 2007.8) were the focus during the early stage. It is worth noting that the genetic research cluster with a large number of emerging keywords shows its importance in future researches.

## Discussion

Our results suggest that the United States, Germany, England and the Netherlands rank higher in terms of numbers of publications, total number of citations and H-index values in the area of CM research, which may be partially explained by some reasons. On one hand, these countries have a high level of both basic and clinical medical research, with specialized doctors and researchers, adequate funding, advanced equipment and well-developed national registration systems. On the other hand, geographically and ethnically, CM is widely considered to be a disease of Caucasian susceptibility, occurring most frequently in the Nordic countries and parts of North America (18). A series of studies based on European cancer registries, especially a recent population-based study which included 41 registries, indicated a higher CM incidence in Northern Europe than that in Southern Europe, in which Norway (0.90 per million population), Finland (0.80), Germany (0.80) and the Netherlands (0.78) exhibited the highest incidence (2, 8, 18, 22–24). On other continents, the incidence was 0.33-0.89 in the United States, 0.25-0.52 in Canada, and 0.60 in Australia (4, 25–27). However, this trend does not mean that non-Caucasians will not suffer from CM. The incidence of Blacks was 0.18, and the incidence of Whites is 2.6 times higher than that of Blacks (28). Increased awareness of CM all around the world leads to the rising publications from outside Europe and North America, such as China (7, 16, 29), India (30), Japan (31) and South Korea (32). However, epidemiological information based on registration data from Asian and African countries is still needed to explore ethnic differences, which is currently only available for South Korea.

Within the top 20 institutions in CM research, nearly half of institutions were from the United States, demonstrating its dominant status in this field. Moreover, a range of institutions (**Figure 4B**) and authors (**Table 1**) have published a large number of papers and made outstanding contributions, reflecting that CM was more studied in the specialized ocular oncology centre. Less experienced ophthalmologists may know little about the treatment of this rare disease, which leads to delays in standard treatment for tumors. Results from two centers suggested that inadequate surgical intervention increases risks of local recurrence and metastasis and that patients who received their first treatment at an ocular oncology center had a better prognosis than those who received their first treatment elsewhere (33, 34). Thus, as researchers suggested, it’s necessary to raise awareness of CM among the public and ocular physicians, and for patients with a suspicious lesions, referring to an experienced ocular oncology center preferably without any prior incisional biopsy, may help achieve better tumor control in patients with CM (7, 33, 34). Remarkably, journals of ophthalmology such as *Ophthalmic Plastic and Reconstructive Surgery*, *British Journal of Ophthalmology*, *American Journal of Ophthalmology* and *Cornea* were the primary journals publishing research on CM. Therefore, future achievements in this field are more likely to be published in these journals.

In recent 25 years, a number of researchers reviewed the tumor characteristics and clinical outcomes at their centers, explored risk factors for poorer prognosis and made tumor management recommendations based on their data and experience. A paper focused on the risk factors for recurrence, exenteration, metastasis, and death in 150 CM patients was published by Shields et al. in 2000, which has been cited 198 times (6). And in 2011, they further expanded the sample size and identified several important prognostic factors, including tumor origin, tumor location, and nodular tumor, which was published in *Ophthalmology*, and was cited 115 times (12). In 2002, Tuomaala et al. found that increasing tumor thickness and local recurrence were associated with increased mortality, which was cited 126 times (8). Esmaeli et al. successively defined histopathological risk factors such as histological ulceration, mitotic figure and vascular invasion and published a series of high-cited papers (9, 19). These studies have contributed to global understanding of this rare disease and guided future research and clinical treatment in this field. Treatment methods are constantly being summarized and innovated. In 1997, Shields et al. introduced a “no-touch” surgical technique to ensure complete removal of the tumor (35). Eight years later, Finger described a novel cryoprobe to freeze large areas with minimal exposure (36). With the continued efforts of researchers, the standard treatment protocol of a wide local excision supplemented by cryotherapy is widely used (1). Radiotherapy, which included brachytherapy and external beam radiation therapy, also plays an important role in the management of CM. Brachytherapy, commonly used as adjuvant therapy after surgical excision, provides good tumor control with visual function preserved in patients with early CM. In a nationwide study in the Netherlands cited 136 times, local recurrences was significantly fewer in patients initially treated with excision and adjuvant brachytherapy than with excision only (3). And external beam radiation therapy is more commonly applied in the patients at a later stage. The benefits and application difficulties of each radiotherapy techniques have been summarized by Spatola et al (37). Topical chemotherapy is also used as adjuvant therapy of surface tumors, and mitomycin C and 5-fluorouracil are commonly used chemotherapy drugs. Researchers are still exploring the substances that effectively inhibit growth of CM to define drugs that may have the potential to add to therapeutic options for local therapy (38).

In recent years, there is an increasing number of publications concentrating on the molecular characteristics in CM. Beadling et al. explored the frequency of *KIT* mutations in various melanoma subtypes, and their results, that *KIT* mutations were detected in 15.6% of mucosal melanomas, 7.7% of CMs, 1.7% of cutaneous melanomas, and 0% of UMs, was published in *Clinical Cancer Research* in 2008 and cited for 440 times (39). Griewank et al. found *BRAF* mutations in 29% of tumors and NRAS mutations in 18%, greatly advancing the understanding of CM pathogenesis (40). To date, the publications have reported that *BRAF* mutations was found in 25%-60% of CM, *NARS* mutations in 18-20% of CM, *NF1* mutations in 20%-33% of CM, and *TERT* promoter mutations in 20%-41% of CM (10, 40–45). Recent work on molecular pathogenesis identified the signatures of UV-induced DNA damage, supporting the role of UV in CM pathogenesis, which is similar to the discovery of cutaneous melanoma (45). More and more literatures showed that CM is closely related to skin melanoma, but it is completely different from intraocular melanoma, which suggests that the therapeutic modalities available for cutaneous melanoma may be effective for CM.

The similarity between CM and cutaneous melanoma offers promise for targeted therapeutic and immunotherapeutic applications of CM, which is rapidly becoming a hot topic in this field. For locally advanced tumors and metastatic diseases, targeted therapy such as BRAF inhibitors and MEK inhibitors shows therapeutic potential. Zaoui et al. tested the tumor suppression effect of BRAF/MEK inhibitors in CM cell-lines, suggesting that targeted therapy may be useful for patients with CM (46). And there are a growing number of case studies on the use of BRAF/MEK inhibitors in BRAF-mutated CM. According to the summary by Zeng et al., more than half of the reported cases achieving disease control (5). In addition, immune checkpoint inhibitors, such as programmed cell death 1 (PD-1) and cytotoxic T lymphocyte antigen-4 (CTLA-4) inhibitors, have been successfully used in advanced cutaneous melanoma and may be effective against CM. Sagiv et al. described the use of PD-1 inhibitors (e.g. nivolumab) in five patients with metastatic CM, and complete response was observed in four patients (47). Finger et al. expanded the use of checkpoint inhibitors to locally advanced and metastatic CM. Responses were observed in all 5 cases, further confirming the effectiveness of immunotherapy (48). Although some valuable case series have been reported, more investigations or clinical trials of targeted therapy and immunotherapy for advanced CM are warranted in the long term.

Our study still has several unavoidable restrictions. Firstly, we only considered publications written in English, and thus, we may have overlooked important research published in other languages. Moreover, in the course of this study, the newer papers could not accumulate a large number of citations, which may affect our conclusion to some extent.

## Conclusion

The study described global trends in conjunctival melanoma research. The United States contributed the most publications and citations followed by Germany, England and the Netherlands. Recent progress can be uncovered in the *Ophthalmic Plastic and Reconstructive Surgery*, *British Journal of Ophthalmology*, *American Journal of Ophthalmology* and *Cornea*. Shields CL, Shields JA, Jager MJ and Finger PT are regarded as good candidates for academic collaboration in CM study. Genetic research, which received much attention currently, reflected the similarity between CM and cutaneous melanoma. Treatments that are successful in cutaneous melanoma, such as targeted therapy and immunotherapy, are gradually becoming a focus of research in CM research.

## Data availability statement

The raw data supporting the conclusions of this article will be made available by the authors, without undue reservation.

## Author contributions

FG, SJ, and SG: Conception and design. WX and LY: Collection and assembly of data. All authors data analysis and interpretation, manuscript writing, final approval of manuscript, accountable for all aspects of the work, contributed to the article, and approved the submitted version. All authors contributed to and revised the final manuscript. All authors contributed to the article and approved the submitted version.

## Funding

This work was supported by the Innovative Research Team of High-Level Local Universities in Shanghai (SHSMU-ZDCX20210901, SHSMU-ZDCX20212802), Shanghai Municipal Science and Technology Project (20JC1411100), Shanghai Jiao Tong University School of Medicine Nursing Development Program (No.Shanghai Jiaoyi [2021]21) and The Science and Technology Commission of Shanghai (20DZ2270800).

## Conflict of interest

The authors declare that the research was conducted in the absence of any commercial or financial relationships that could be construed as a potential conflict of interest.

## Publisher’s note

All claims expressed in this article are solely those of the authors and do not necessarily represent those of their affiliated organizations, or those of the publisher, the editors and the reviewers. Any product that may be evaluated in this article, or claim that may be made by its manufacturer, is not guaranteed or endorsed by the publisher.
